# The thickness of the crystal mush on the floor of the Bushveld magma chamber

**DOI:** 10.1007/s00410-017-1423-4

**Published:** 2017-11-16

**Authors:** Marian B. Holness, R. Grant Cawthorn, James Roberts

**Affiliations:** 1 Department of Earth Sciences, University of Cambridge, Downing Street, Cambridge, CB2 3EQ UK; 2 School of Geosciences, University of the Witwatersrand, PO Wits, Johannesburg, 2050 South Africa; 3 Department of Geology, University of Pretoria, Private Bag X20, Hatfield, Pretoria, 0028 South Africa

**Keywords:** Crystal mush, Layered intrusion, Bushveld Intrusion, Microstructure, Dihedral angle

## Abstract

The thickness of the crystal mush on magma chamber floors can be constrained using the offset between the step-change in the median value of dihedral angles formed at the junctions between two grains of plagioclase and a grain of another phase (typically clinopyroxene, but also orthopyroxene and olivine) and the first appearance or disappearance of the liquidus phase associated with the step-change in median dihedral angle. We determined the mush thickness in the Rustenburg Layered Suite of the Bushveld Complex at clinopyroxene-in (in Lower Main Zone) and magnetite-in (in Upper Zone). We also examined an intermittent appearance of cumulus apatite in Upper Zone, using both the appearance and disappearance of cumulus apatite. In all cases, the mush thickness does not exceed 4 m. These values are consistent with field observations of a mechanically rigid mush at the bases of both magnetitite and chromitite layers overlying anorthosite. Mush thickness of the order of a few metres suggests that neither gravitationally-driven compaction nor compositional convection within the mush layer is likely to have been important processes during solidification: adcumulates in the Bushveld are most likely to have formed at the top of the mush during primary crystallisation. Similarly, it is unlikely either that migration of reactive liquids occurs through large stretches of stratigraphy, or that layering is formed by mechanisms other than primary accumulation.

**Electronic supplementary material:**

The online version of this article (10.1007/s00410-017-1423-4) contains supplementary material, which is available to authorized users.

## Introduction

The thickness of the crystal mush on the floor of magma chambers, defined as the vertical interval separating an essentially rigid assemblage of cumulus and intercumulus grains within which any remaining liquid is trapped and immobile, and the interface with the overlying bulk magma, is of interest as it is the physical properties of this mush, and the way it forms during solidification, that control the efficiency of fractionation. To date, there is a wide range of ideas concerning the thickness of mush layers, ranging from several hundred metres to essentially zero thickness. As a very general approximation, estimates of mush thickness based on theoretical models of the behaviour of layered intrusions (e.g. Irvine 1980; Tait and Jaupart 1992; McKenzie 2011) tend to be larger than those based on field observations (e.g. Thompson and Patrick 1968; Holness and Winpenny 2009; Maier et al. 2013; Cawthorn 2015; Latypov et al. 2015).

Recently, it was argued that the thickness of the crystal mush can be determined at the specific stratigraphic position recording the saturation of the bulk magma in a new liquidus phase (or when the bulk magma lost a liquidus phase). This is done by measuring the stratigraphic distance between the arrival (or disappearance) of the liquidus phase in the primocryst assemblage and the step-change in dihedral angle associated with the changing fractional contribution of latent heat to the enthalpy budget (Holness et al. 2017a). The base of the mush is marked by the step-change in dihedral angle (which effectively marks the point at which solidification is almost complete), whereas the top of the mush is marked by the change in the liquidus assemblage.

In this contribution, we apply this dihedral angle method to three widely spaced horizons in the stratigraphy of the Bushveld Complex of South Africa, and demonstrate that the floor mush was only a few metres thick. These results are consistent with field observations that suggest a generally thin mush throughout the stratigraphy of the intrusion.

## Mush thickness from dihedral angle steps

The geometry of clinopyroxene–plagioclase–plagioclase three-grain junctions varies systematically within mafic intrusions, governed by the response of plagioclase growth faces to changes in crystallisation kinetics (Holness 2015). In essentially unfractionated bodies such as dolerite sills, the median dihedral angle, Θ_cpp_, varies smoothly and symmetrically, with higher values in the centre of the sill compared to the edges, correlating with crystallisation timescale (Holness et al. 2012a). In fractionated bodies such as layered intrusions, Θ_cpp_ is constant over large stretches of stratigraphy, with step-wise changes associated with changes in the number of phases on the liquidus. An increase in the number of phases results in a step-wise increase in Θ_cpp_, whereas Θ_cpp_ decreases when the number of liquidus phases decreases (Holness et al. 2013).

The geometry of clinopyroxene–plagioclase–plagioclase three-grain junctions in most gabbros and dolerites records processes active during solidification. The creation of three-grain junctions is a gradual process, with the progressive filling of increasingly narrow pores as solidification proceeds: all clinopyroxene–plagioclase–plagioclase dihedral angles are formed when the rock is > 90 vol.% solidified (Holness et al. 2012b). Because the melt topology in solidifying gabbros is unlikely to be in textural equilibrium (Holness et al. 2012b), there is a finite porosity (the percolation threshold) below which the remaining melt no longer forms an interconnected network. Cheadle et al. (2004) showed that the percolation threshold occurs at 8–11 vol.% for non-texturally equilibrated systems, suggesting that once solidification has proceeded sufficiently to result in the completion of all dihedral angles (at least in gabbros which are not undergoing deformation) any remaining liquid is essentially immobile.

**Fig. 1:**
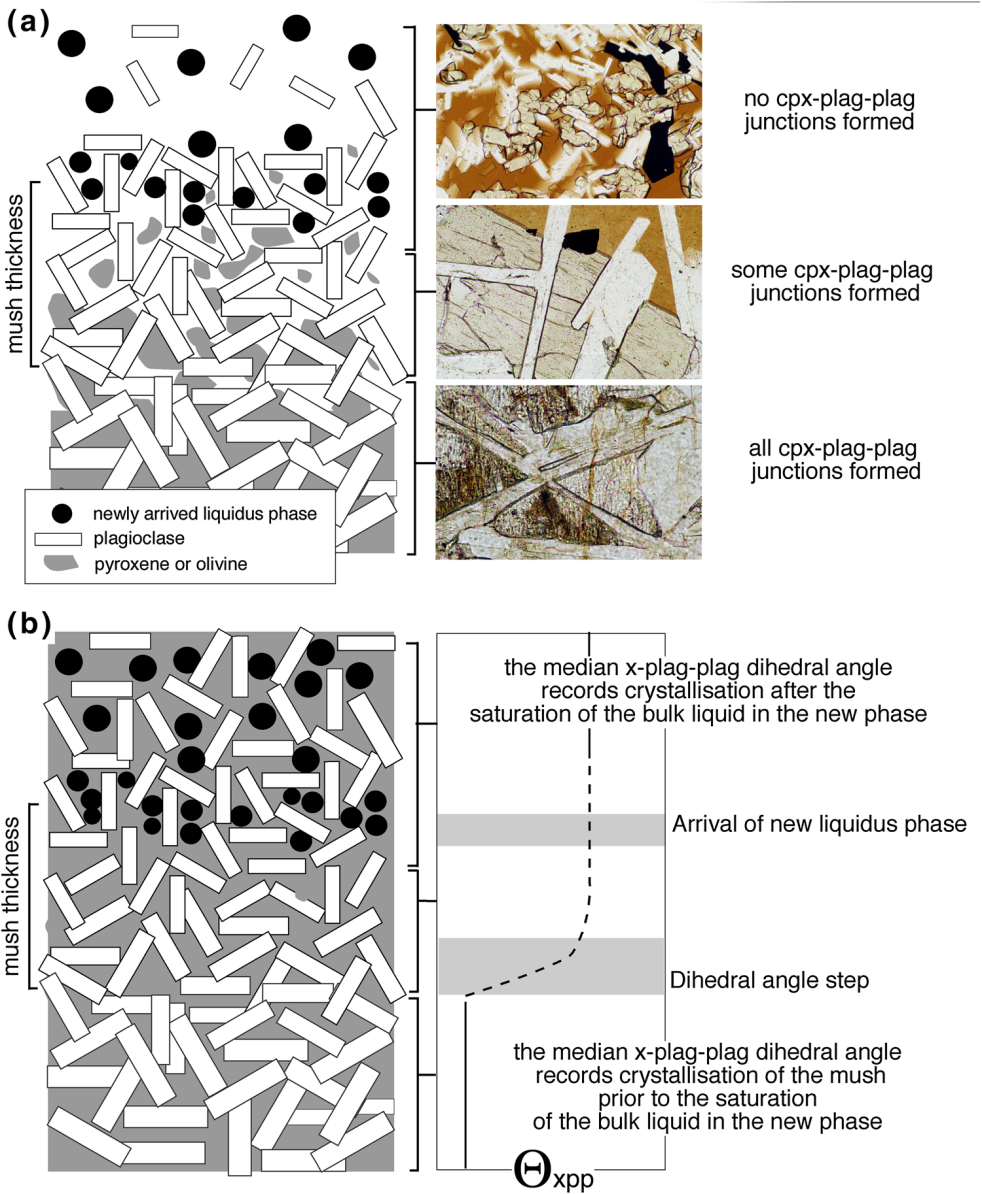
**a** A highly schematic cartoon showing the structure of a gabbroic mush layer at the moment the overlying bulk magma saturates in a new liquidus phase. The base of the mush is the horizon at which all dihedral angles between two grains of plagioclase (denoted by the white rectangles) and a grain of either pyroxene or olivine (denoted by the grey regions) are formed. The top of the mush is defined by the horizon at which the new liquidus phase forms abundant and homogeneously distributed grains. To the right are photomicrographs illustrating the extent of solidification through the upper crust of the Kilauea Iki lava lake: all possible three-grain clinopyroxene–plagioclase junctions are formed at the base of the mush, whereas almost none have been created near the top. **b** A cartoon showing the fully solidified mush zone, with an illustration of the variation through the stratigraphy of the fully solidified cumulate of the median value of dihedral angle formed at the junctions of two grains of plagioclase (p) and one grain of either pyroxene or olivine (x), Θ_xpp_

Mush thickness can be constrained at specific stratigraphic horizons using the step-wise change in Θ_cpp_ caused by changes in fractional latent heat (Holness et al. 2007a, b; Holness et al. 2009; Morse 2011). The first appearance (or disappearance) of the relevant primocrysts in the cumulate stratigraphy marks the top boundary of the mush at the instant the bulk magma becomes saturated with (or loses) the new phase (Fig. 1a). The base of the Θ_cpp_ step marks the point at which the dihedral angle changes from the value associated with the old liquidus assemblage to that associated with the new liquidus assemblage (Fig. 1b), triggered by a change in the growth behaviour of plagioclase in response to a change in the local latent heat contribution to the enthalpy budget.

In the case of step-changes associated with an increase of the number of liquidus phases, the base of the Θ_cpp_ step marks the level at which all possible clinopyroxene–plagioclase–plagioclase three-grain junctions were formed at the moment the bulk liquid above the mush became saturated in the new liquidus phase (Fig. 1). All possible angles will be formed in solidifying orthocumulates (i.e. those cumulates initially containing abundant interstitial liquid, similar to the basalts of the Kilauea Iki crust) when all but ~ 10 vol.% of the interstitial liquid has solidified (Holness et al. 2012b); in rocks with more adcumulate character, dihedral angle formation will be complete only when the rock is closer to 100% solidification. In all cases, the base of the step corresponds to the horizon at which the volume of remaining liquid had dropped below the permeability threshold (c.f. Cheadle et al. 2004), with retention and immobilisation of any remaining liquid below this stratigraphic horizon. The more adcumulate the rock is, the more closely the stratigraphic position of the dihedral angle step corresponds to the horizon at which the mush is fully solidified. The base of the step, therefore, provides a marker for the base of the crystal mush, and the mush thickness is recorded by the distance from the base of the step to the first appearance of the new liquidus phase in the cumulates (Fig. 1; Holness et al. 2017a).

While it is not possible to change the sequence in which the various textural and compositional markers appear in the stratigraphy of the final, fully solidified cumulate, the spacing between the markers can be altered during solidification by melt expulsion (and volume decrease) due to gravitationally-driven compaction or shearing. The primary porosity of an accumulating gabbroic mush depends on grain shape and orientation, and the extent to which grains form chains and clusters. A mechanically coherent framework can form with porosities of 60–75 vol.% (e.g. Campbell et al. 1978; Philpotts and Dickson 2000). Since the final porosity may be only a few vol.%, the *maximum* possible effect of melt expulsion is to reduce the apparent mush thickness recorded by microstructures in fully solidified cumulates by a factor of ~ 0.25.

## Analytical methods

True dihedral angles were measured using a 4-axis Leitz universal stage mounted on a James Swift monocular optical microscope, with a UM32 Leitz objective and a × 10 eyepiece. Since the process controlling the magnitude of the clinopyroxene–plagioclase–plagioclase dihedral angle is the response of plagioclase to changes in crystallisation rates (Holness 2015), the median dihedral angle measured at junctions between two plagioclase grains and a grain of orthopyroxene or olivine is indistinguishable from that at junctions involving clinopyroxene. The coarse grain size and microstructure of many of the UZ samples we examined meant that a single thin section may not contain sufficient numbers of clinopyroxene–plagioclase–plagioclase junctions: we therefore measured dihedral angles at junctions with clinopyroxene where possible but augmented the datasets with measurements at olivine–plagioclase–plagioclase junctions in the region of apatite-in, and with measurements at orthopyroxene–plagioclase–plagioclase junctions in the region of magnetite-in. We report here the median value of the clinopyroxene–plagioclase–plagioclase dihedral angle, Θ_cpp_, for Lower Main Zone, the pyroxene–plagioclase–plagioclase dihedral angle, or Θ_ppp_, for the lower part of Upper Zone, and a mixture of olivine–plagioclase–plagioclase and clinopyroxene–plagioclase–plagioclase, denoted Θ_xpp_, for the upper part of Upper Zone.

The median value of a population of angles can be determined satisfactorily with only 25 measurements (Riegger and Van Vlack 1960), although reduction of the uncertainty on the median to < ± 2–4° generally requires more than 50 measurements for those samples with a wide range of true angles (Holness 2010). For each sample, between 30 and 60 individual measurements were made (Table 1): this necessitated making duplicate or triplicate thin sections for some particularly coarse-grained samples. Quoted uncertainties are the 2*σ* confidence intervals about the median calculated according to the method of Stickels and Hücke (1964).

**Table 1:** Data for the Rustenburg Layered Suite

Sample	Mineral	*n*	Median angle
Lower Main Zone (sampled by Lonmin drill core SL12)			
SL12 1529.80	Clinopyroxene	30	82.5 ± 3.5
SL12 1432.95	Clinopyroxene	30	94.5 ± 3
SL12 1425.55	Clinopyroxene	30	84 ± 3
SL12 1395.77	Clinopyroxene	30	85.5 ± 3
SL12 1388.49	Clinopyroxene	30	86 ± 4
SL12 1380.92	Clinopyroxene	40	85 ± 4
SL12 1380.50	Clinopyroxene	50	86.5 ± 3.5
SL12 1379.70	Clinopyroxene	30	84 ± 4
SL12 1379.20	Clinopyroxene	50	96 ± 2.5
SL12 1377.15	Clinopyroxene	50	94.5 ± 2
SL12 1373.60	Clinopyroxene	50	95.5 ± 2.5
SL12 1366.39	Clinoyroxene	40	95 ± 2.5
SL12 1335.93	Clinopyroxene	30	98 ± 3
SL12 1305.39	Clinopyroxene	30	96.5 ± 4
Lower Upper Zone (sampled by drill core BK2)			
2W 225.0	Clinopyroxene and orthopyroxene	60	85 ± 1.5
2W 211.0	Clinopyroxene and orthopyroxene	40	84 ± 2
2W 201.0	Clinopyroxene and orthopyroxene	50	85 ± 2
2W 200.5	Clinopyroxene and orthopyroxene	40	87 ± 3
2W 200.0	Clinopyroxene and orthopyroxene	30	91 ± 2.5
2W 199.0	Clinopyroxene and orthopyroxene	50	91 ± 2.5
2W 196.0	Clinopyroxene and orthopyroxene	50	89.5 ± 3
2W 177.5	Clinopyroxene and orthopyroxene	50	91.5 ± 3
Upper Upper Zone (sampled by drill core BK1)			
1W 1583.4	Clinopyroxene and olivine	50	91 ± 2
1W 1578.0	Clinopyroxene and olivine	50	90 ± 3
1W 1575.54	Clinopyroxene and olivine	50	90 ± 3
1W 1575.0	Clinopyroxene And olivine	50	94 ± 2
1W 1574.5	Clinopyroxene and olivine	50	95.5 ± 2
1W 1573.5	Clinopyroxene and olivine	50	95.5 ± 2
1W 1572.0	Clinopyroxene and olivine	50	95.5 ± 2
1W 1550.7	Clinopyroxene and olivine	60	96.5 ± 2
1W 1549.3	Clinopyroxene and olivine	50	95.5 ± 2
1W 1548.5	Clinopyroxene and olivine	40	96 ± 3
1W 1546.8	Clinopyroxene and olivine	40	96 ± 2
1W 1546.35	Clinopyroxene and olivine	50	93 ± 3
1W 1545.97	Clinopyroxene and olivine	30	90 ± 3
1W 1545.14	Clinopyroxene and olivine	40	90 ± 3
1W 1544.6	Clinopyroxene and olivine	50	90.5 ± 3
1W 1543.96	Clinopyroxene and olivine	50	90 ± 2

Samples beginning SL12 are from the Lower Main Zone, with depths in core (in metres) indicated by the sample number. Samples beginning BK1 and BK2 are from Bierkraal drill cores BK1 and BK2, respectively, with sample numbers indicating depth in core in metres. The number of observations is given by *n*; mineral gives the name of the mineral(s) forming the three-grain junction with plagioclase. Uncertainties are calculated according to Stickels and Hücke (1964)

## Geological setting

**Fig. 2:**
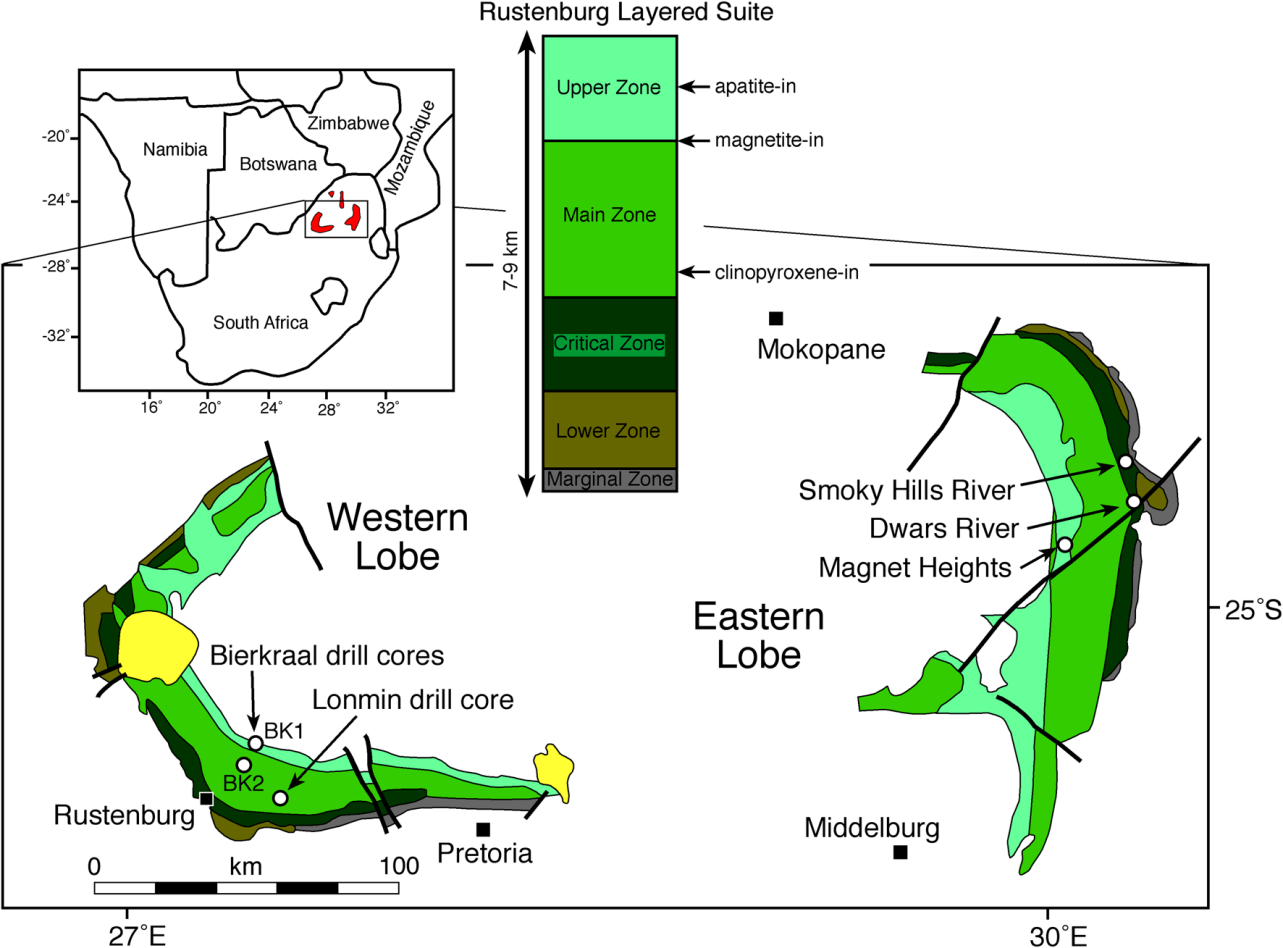
A simplified geological map of the Bushveld Complex, with a schematic stratigraphy through the Rustenburg Layered Suite (after Pebane and Latypov 2017). The yellow colour denotes later alkaline intrusions

The ~ 2.06 Ga Bushveld Complex of South Africa is the largest known mafic layered intrusion on Earth and outcrops in two main arcuate sections (Fig. 2). It is thought that this outcrop pattern is the result of isostatic subsidence of an originally continuous sill-like sheet (Du Plessis and Kleywegt 1987; Cawthorn et al. 1998; Webb et al. 2004). Field evidence suggests deformation of the intrusion (on scales of the order of tens of km), including updoming of the underlying country rock, may have been episodic, beginning shortly after emplacement and continuing after the intrusion was fully solidified (e.g. Clarke et al. 2005; Letts et al. 2009; Maier et al. 2013).

The stratigraphy of the ultramafic to mafic Rustenburg Layered Suite of the Bushveld Complex is subdivided into Marginal, Lower, Critical, Main and Upper Zones (Fig. 2), although the precise position of the boundaries between these various subdivisions has been the subject of debate (Kruger 1990). It intruded as a series of magma pulses, with a major recharge event occurring within the Main Zone, about two-thirds of the way up the stratigraphy. The incoming magma of this recharge event mixed with the resident magma to form an essentially compositionally uniform body. That this recharge represented the last influx into the chamber is suggested by von Gruenewaldt (1973), Molyneux (1974) and Kruger et al. (1987), although recent work argues for further influxes throughout Main (Hayes et al. 2017) and Upper Zone (Yuan et al. 2017).

The simplified order of appearance of minerals through the entire ~ 8 km of stratigraphy is olivine, chromite, orthopyroxene, (olivine out), plagioclase, (chromite out), clinopyroxene, magnetite, (ferrian) olivine (orthopyroxene out), apatite. Many of these phase changes are cyclic, rhythmic or at least not continuously present: this behaviour is most obvious in the Upper Zone, which is subdivided into UZa, UZb and UZc on the basis of the re-appearance of cumulus olivine and the arrival of cumulus apatite. In this contribution, we are concerned with the first appearance of clinopyroxene, magnetite and apatite as cumulus phases.

Clinopyroxene first appears as a cumulus mineral near the base of Main Zone (Fig. 2), but the precise stratigraphic position of its arrival is not well known. In the eastern limb of the Bushveld, von Gruenewaldt (1973) described the lower 1200 m of Main Zone as norite on the basis that the proportion of orthopyroxene exceeded clinopyroxene. However, the modal data he presented indicate cyclic variation in the relative proportions of the two pyroxenes over several hundred metres of that stratigraphy. The stratigraphy in the western limb is generally derived from the abundant drill core created during exploration for the platiniferous Merensky Reef. Mitchell (1990) reported two noritic units separated by anorthosite at the base of the Main Zone, totalling 300 m of stratigraphy, overlain by four gabbronoritic units. To evaluate the transition from Critical to Main Zone, Mitchell and Manthree (2002) obtained 36 samples from 100 m of borecore across that boundary (mainly in Main Zone), averaging 1 sample every 3 m. Clinopyroxene exceeded 11% in only one sample.

**Fig. 3:**
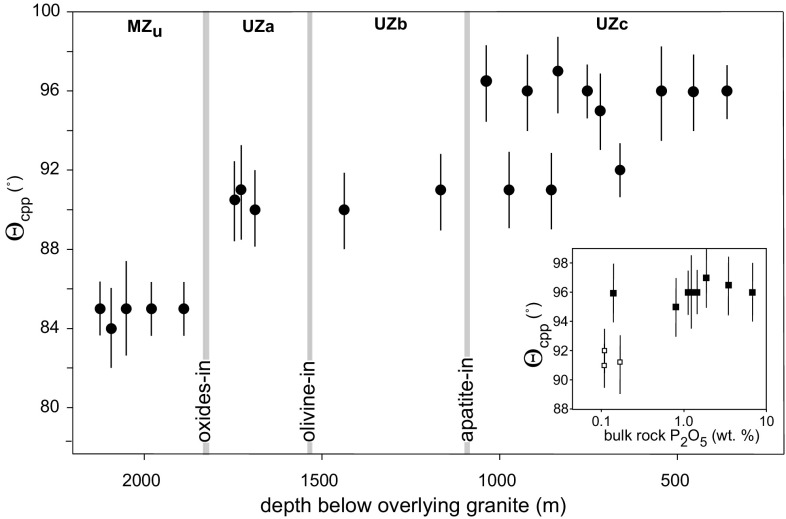
The stratigraphic variation of Θ_cpp_ from the upper part of Main Zone through the Upper Zone. Note the step-wise changes in Θ_cpp_ associated with the increase in number of phases in the liquidus assemblage (magnetite-in and apatite-in), while the arrival of olivine (which is replacing orthopyroxene in the liquidus assemblage and is thus not associated with a change in the number of liquidus phases) is not associated with a step. The bimodal behaviour of Θ_cpp_ in UZc is associated with the cyclic presence of cumulus apatite (see the inset, which shows the correlation between bulk rock P_2_O_5_ and Θ_cpp_). Data from Holness et al. (2013)

Cumulus magnetite first appears just below the 2 m thick magnetitite known as the Main Magnetitite Layer and its first appearance defines the marker for the base of Upper Zone (marking the base of UZa, Fig. 3). A striking feature of Upper Zone is the presence of ~ 25 distinct magnetitite layers, commonly with sharp bases but with tops that grade into anorthosite. Holness et al. (2013) documented a step-wise increase in Θ_cpp_ associated with the arrival of cumulus magnetite (Fig. 3).

Following the work of Cawthorn and Walsh (1988) who report the cyclic appearance and disappearance of cumulus apatite, a detailed geochemical and petrographic study of the uppermost 2.1 km of layered mafic rocks sampled by three boreholes drilled at Bierkraal in the western limb of the intrusion detected at least nine distinct cycles marked by reversals in mineral composition and the intermittent presence of cumulus apatite and olivine (Tegner et al. 2006). These cycles are attributed to periodic mixing driven by density inversion of a compositionally stratified magma body, triggered for at least two of these cycles by the crystallisation of thick magnetitite layers (Tegner et al. 2006). The cyclic nature of the appearance and disappearance of cumulus apatite is reflected in the bimodal value of Θ_cpp_ through UZc, with low values indicative of no cumulus apatite (Fig. 3, Holness et al. 2013).

## Field observations of mush behaviour

The stratigraphy of the Rustenburg Layered Suite is remarkable for the large number of laterally extensive, almost monomineralic, layers of oxide, either chromite in Critical Zone or magnetite in Upper Zone. An examination of the response of the cumulates underlying oxide layers is of particular interest when considering field evidence for mush behaviour because of the extreme gravitational instability inherent in placing oxides (density ~ 5.1–5.2 g cm^−3^) on top of plagioclase-rich material (density ~ 2.7 g cm^−3^). If the anorthosite footwall were poorly consolidated (i.e. if the mush were thick), one might expect to see evidence for load structures and the squeezing out of interstitial liquid, akin to those observed in sequences of sedimentary rocks. If the anorthosite was well consolidated (i.e. if the mush was thin), such evidence for loading would not be present.

**Fig. 4:**
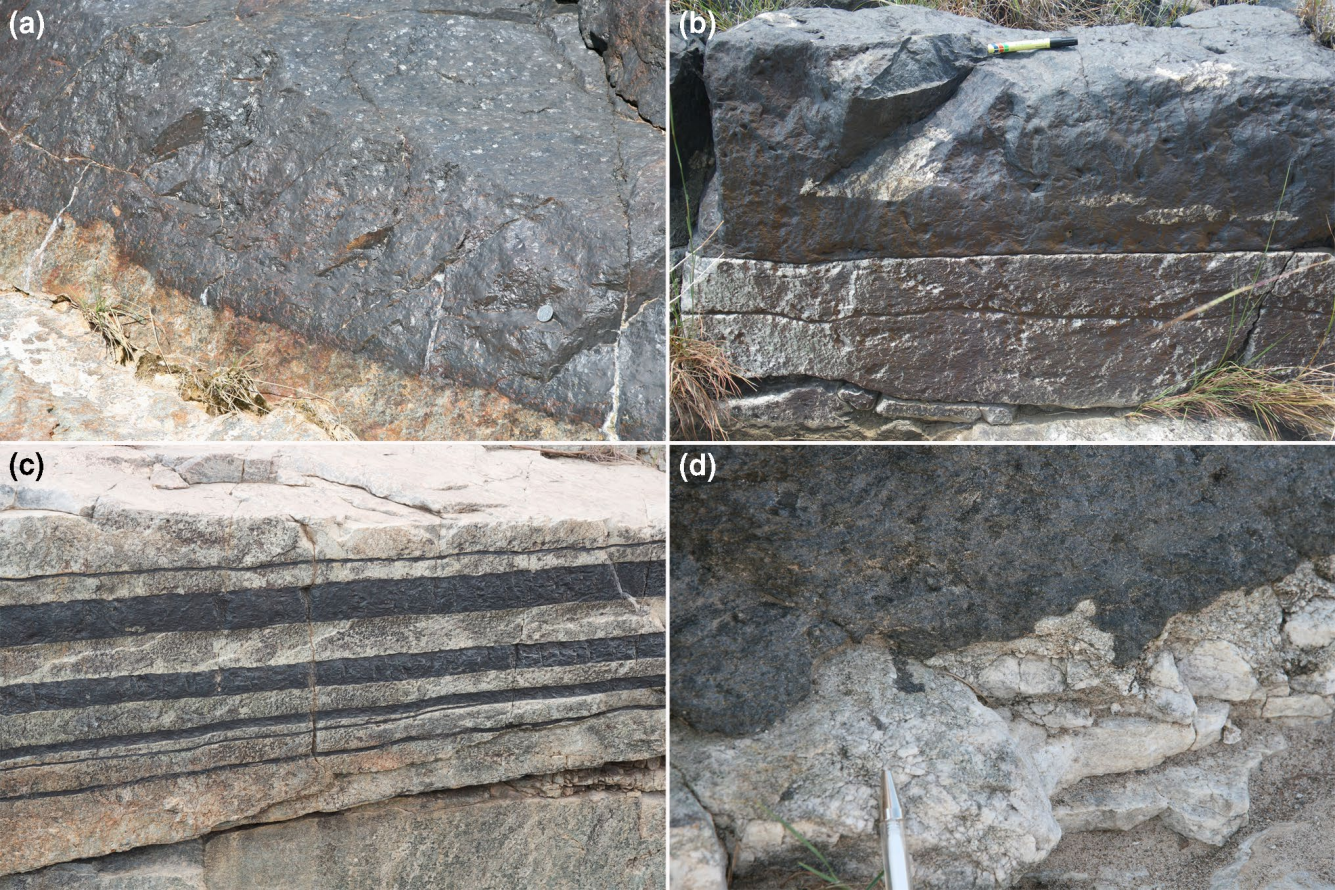
Photographs of oxide layers in the Bushveld Complex. **a** The base of the Main Magnetitite Layer at Magnet Heights (see Fig. 2 for location). Note the almost perfectly smooth contact with the underlying anorthosites, with little evidence of gravitational loading. Coin for scale near right of image. **b** The base of a chromitite layer (these layers are collectively known as UG1) at Dwars River (see Fig. 2 for location). Note the elongate fragments of anorthosite aligned parallel with the layering and the planar and well-defined contact between the chromitite layer and the underlying anorthosite. Pen for scale. **c** Multiple continuous and thin chromitite layers at Dwars River. The thickest of the chromitite layers is ~ 10 cm thick. **d** The base of the Main Magnetitite Layer at Magnet Heights is locally irregular. The scale of the irregularities is of the order tens of centimetes (note pen in the image). Their origin is enigmatic: they may be localised load structures or evidence of thermochemical erosion (c.f. Latypov et al. 2015)

The lower contacts of both the chromitite and the magnetitite layers are generally very sharply defined, are commonly planar at outcrop scale (e.g. Pebane and Latypov 2017), and show little evidence for gravitational loading (Fig. 4) [with exceptions illustrated by Maier et al. (2013) and Mungall et al. (2016)]. Fluid escape structures at the base of oxide layers are only rarely reported (e.g. Maier et al. 2013), indicating little evidence of upward flow of over-pressured interstitial liquid driven from the underlying anorthosites. A detailed study of a rare, laterally extensive, region of exposed surface at the base of the Upper Group 2 (UG2) chromitite demonstrated that chromite-filled depressions in the top surface of the underlying anorthosite are separated by planar regions of interface, incompatible with formation by gravitational loading (Van der Merwe and Cawthorn 2005). Such features can only have formed if the underlying anorthosite was mechanically strong when the very dense oxide layer was deposited. This is supported by the presence of well-defined anorthosite fragments within chromite layers (Fig. 4b; Latypov et al. 2015) [although these anorthosite fragments may have been derived from elsewhere, Pebane and Latypov (2017) provide evidence of a local source for some].

**Fig. 5:**
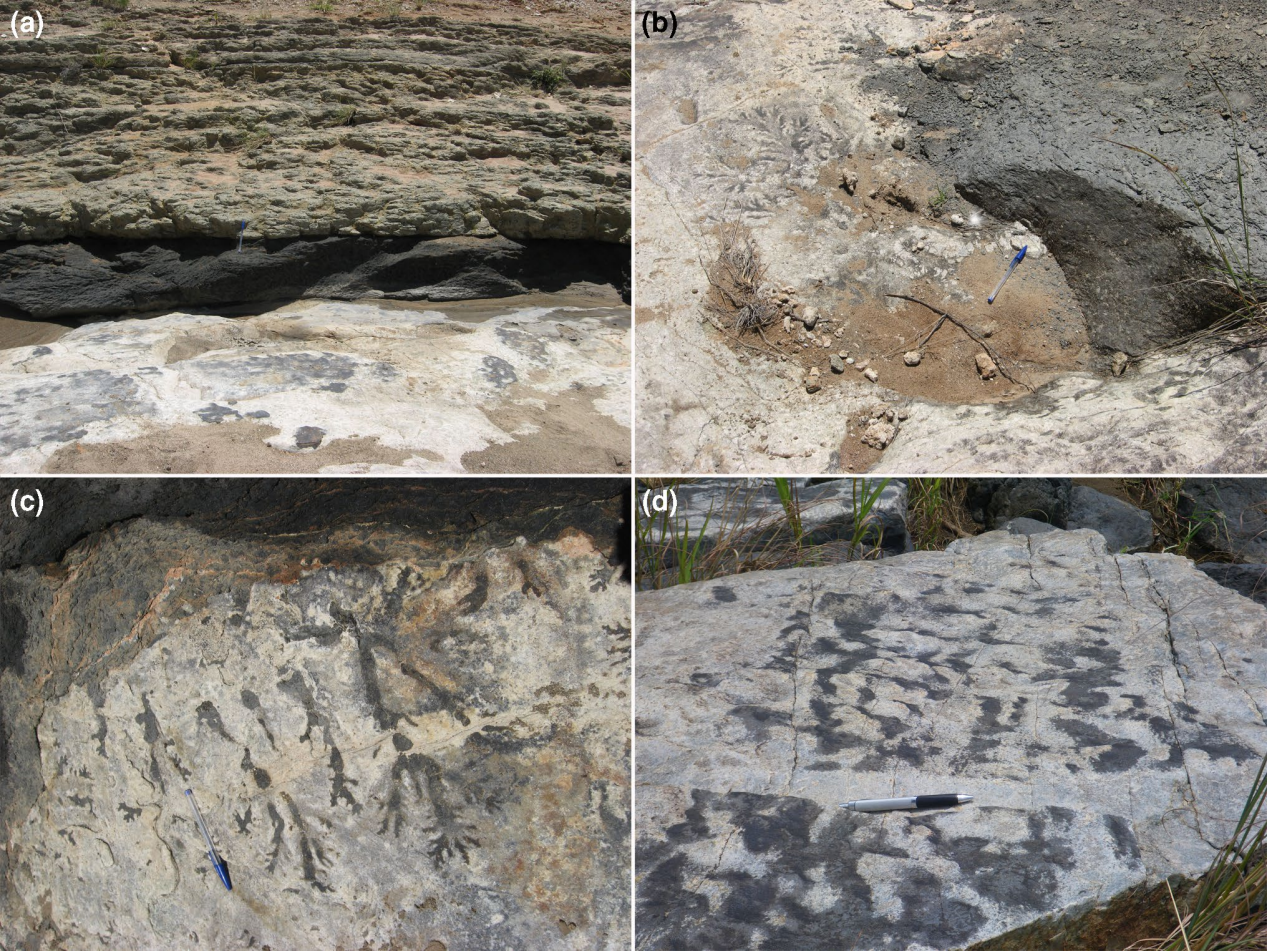
Photographs of localised evidence of mechanically coherent anorthositic mush. **a** In the bed of the Smokey Hills River on Maandagshoek Farm (see Fig. 2 for location), the stratigraphy comprises a lower, erosion-resistant anorthosite, overlain by a ~ 40 cm thick chromitite layer (UG3) which itself is overlain by a green pyroxenite. **b** The exposed top surface of the anorthosite at Smokey Hills reveals submetre-scale potholes which are filled with the UG3 chromitite. The sides of these potholes are locally overhanging (not shown). Pen for scale. **c** The edges of the Smokey Hills potholes are marked by a series of branching shallow channels filled with chromite: these are highly reminiscent of erosion of the upper surface of the anorthosititic mush forming drainage patterns around the potholes (pen for scale). **d** The upper surface of anorthosite at Dwars River (see Fig. 2 for location) locally shows evidence of discontinuous elongate depressions that are highly reminiscent of ripples, again suggestive of the local reworking of a generally mechanically coherent mush layer (pen for scale)

The contact between the base of chromite layers in Critical Zone and the underlying anorthosite layers (Fig. 5a) is locally characterised by metre-scale erosional features known as potholes (Fig. 5b). The potholes may have overhanging walls, and the anorthosite surface is locally marked by channels suggestive of erosional flow into the potholes (Fig. 5b, c). These features, including evidence for truncation of primary igneous layering in the walls (Maier et al. 2013; Latypov et al. 2015) and lack of disruption of layering under the potholes (Van der Merwe and Cawthorn 2005), and evidence of the apparent erosion of early formed oikocrysts during pothole formation (Latypov et al. 2015), point to mechanical coherence and strength of the anorthosite when it was eroded prior to the deposition of the chromite. Further evidence of a mechanically coherent anorthositic mush is provided by the local development of weakly linear and discontinuous undulations of the anorthosite–chromitite boundary that are remarkably like ripples, suggestive of erosional reworking of the surface of the mush (Fig. 5d).

**Fig. 6:**
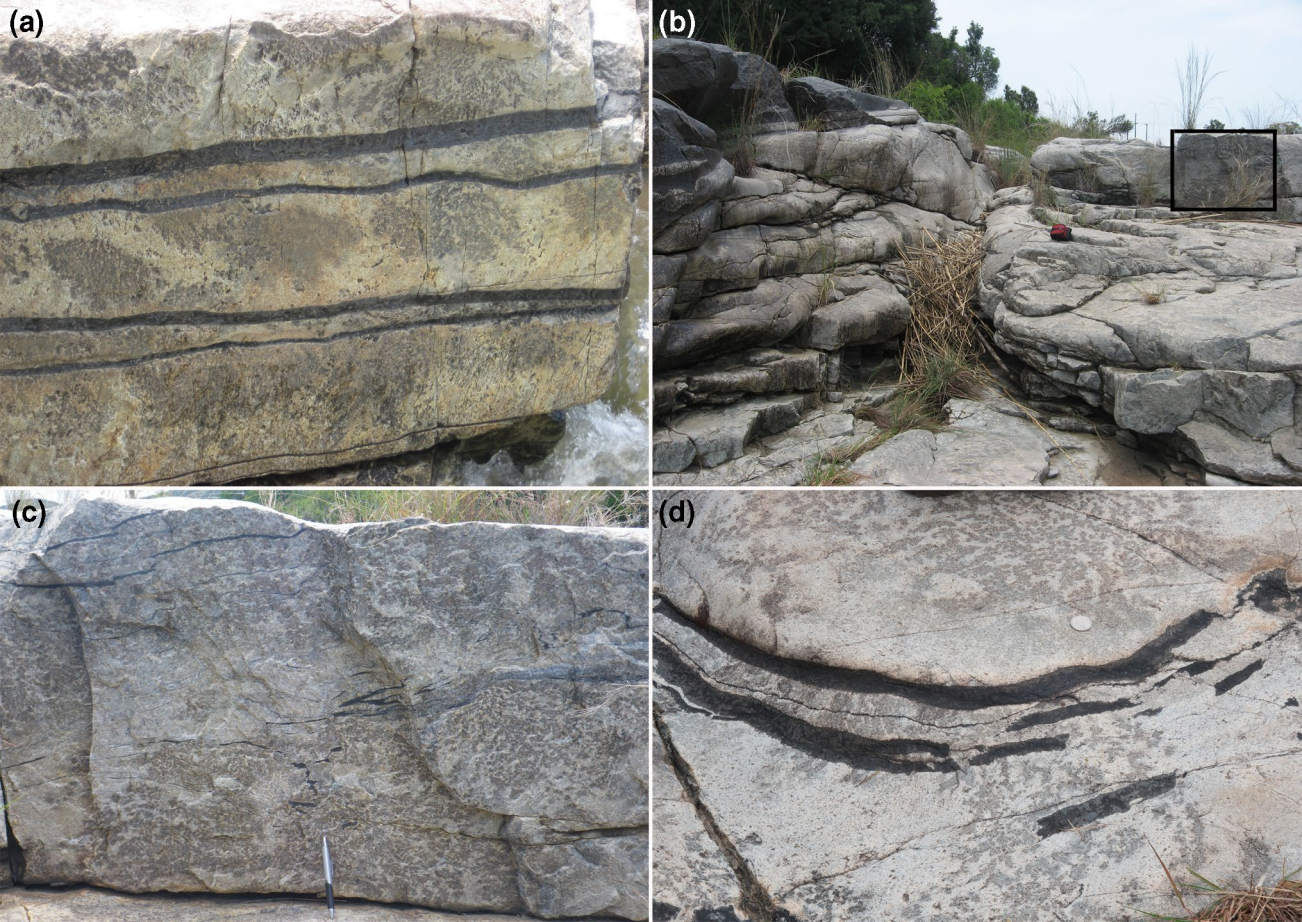
Photographs showing localised evidence of mush deformation. **a** Thin chromitite layers (UG1) at Dwars River (see Fig. 2 for location) have sharp top and bottom contacts against the anorthosite but are locally non-planar. Note the slight undulations spatially associated with dm-scale orthopyroxene oikocrysts, suggestive of limited compaction post-dating oikocryst growth. **b** Large-scale view of Dwars River locality showing the planar and sharply defined lower surface of a dm-scale chromitite layer (to the left) The area of anorthosite shown in close-up in **c** is shown by the black box. Red camera case for scale in centre of field of view. **c** Close-up of area outlined in **b** showing ductile deformation and disruption of cm-scale chromitite layers. Pen for scale. **d** Brittle deformation of cm-scale chromitite layers. Layers with this style of deformation occur immediately below the camera case in **b**, although this particular example (photographed some tens of metres away) shows the phenomenon more clearly than the layer in the field of view depicted in **b**. Note the coin for scale

The evidence presented so far simply attests to the mechanical strength of the anorthosite and is at least compatible with a thin mush. At Dwars River, a well-known locality in Critical Zone noted for its well-defined and alternating layers of chromitite and anorthosite [Fig. 2; see Pebane and Latypov (2017) for detailed field description of this locality], the extensive exposure provides evidence for the stratigraphic interval over which the mush rheology varies and therefore an indication of mush thickness. The thinner chromitite layers that underlie the Upper Group 1 (UG1) chromitite are locally deflected around large (10–20 cm diameter) orthopyroxene oikocrysts (Fig. 6a), suggestive of limited compaction immediately post-dating oikocryst nucleation and growth [although Pebane and Latypov (2017) suggest that oikocryst growth locally post-dated ductile deformation and disruption of the chromitites].

Evidence is provided of changes in rheological behaviour over a few metres of stratigraphy (Fig. 6b), with the thick UG1 chromitite layer characterised by a planar base overlying several metres of anorthosite in which thin chromitite layers are highly disturbed in an apparently ductile manner (Fig. 6c), whereas chromitite layers only 2–3 m below are deformed in a brittle manner [Fig. 6d; see Pebane and Latypov (2017) for further examples]. Although we have no indication of time-scales for this recorded variation, it is suggestive of an anorthosite mush of no more than a few metres thick, with a brittle response to disturbance near the base and a more ductile response near the top. Before the deposition of the overlying thick UG1 chromitite layer, the mush then became sufficiently mechanically coherent to prevent the formation of gravitational loading structures at this particular anorthosite–chromitite boundary. These observations are consistent with the work of Mukherjee et al. (2017) who argue for a mechanically coherent anorthosite 1.5 m below the top of the mush at the moment of formation of the UG1 chromitite.

**Fig. 7:**
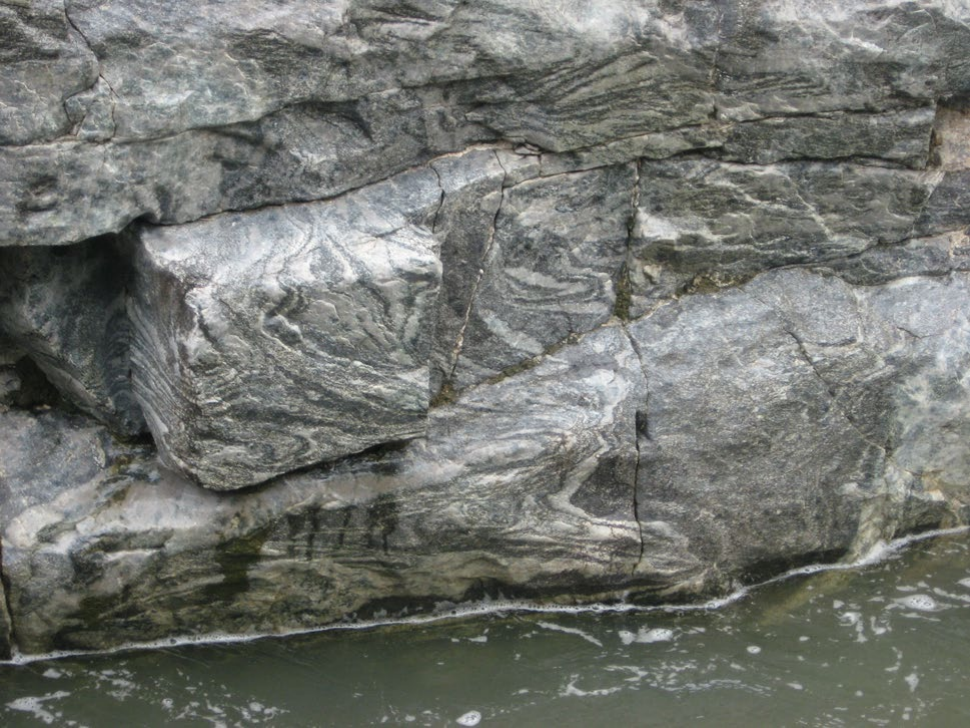
Highly disrupted and distorted layering in the anorthosite underlying UG1 at Dwars River (see Fig. 2 for location). This disrupted package of cumulates is 3 m thick (Cawthorn 2015) although the lateral scale of the disruption is not known

That the mush rheology, and hence thickness of the high porosity region of the mush, was temporally highly variable in the Dwars River locality is demonstrated by the alternation of the planar interbedded anorthosite/chromitite layering described earlier with horizons typified by extensively disrupted and folded layering (Cawthorn 2015). These occur both below [Fig. 7; see also Fig. 201 of Wager and Brown (1968)] and above the stratigraphy illustrated in Fig. 6. Such highly contorted layering demonstrates that deformation and slumping of fluid-rich mush was episodic, with intervening periods of quiescence, suggestive of earthquake-triggered deformation events (Cawthorn 2015) possibly associated with syn-magmatic diapiric updoming of the underlying country rocks (Clarke et al. 2005).

## Choice of samples for petrographic analysis

### Lower Main Zone

Borecore (SL12) was provided by Lonplats mining company, from their mine at Mooinooi, 30 km east of Rustenburg in the western Bushveld (Fig. 2). Over 500 m of stratigraphy was sampled, extending upwards from the Merensky Reef (the base of which is located at 1567.75 m depth in the core). The base of the Giant Mottled Anorthosite (a marker used to designate the base of the Main Zone) occurs at a depth of 1546.45 m in the core. The sample spacing was generally 7–8 m except in regions of interest, in which it was reduced to ~ 0.5 m.

### Upper Zone

Three deep bore holes were drilled by the Geological Survey of South Africa, collared NE of Rustenburg in the western part of the Bushveld Complex (Fig. 2). They provide a continuous profile through the top of the Main Zone and entire Upper Zone (Kruger et al. 1987). The average dip of the layering is 24°, whereas actual depths in the core are reported here. The Bierkraal borehole BK1 was collared in Bushveld granite overlying the layered mafic rocks, and encounters the arrival of cumulus apatite. Borehole BK2 extends from near the base of the Upper Zone and encounters the arrival of cumulus magnetite in Main Zone.

The base of the Main Magnetitite Layer appears at a depth of 171 m in drill core BK2 (Kruger et al. 1987, their Fig. 2). To investigate the arrival of cumulus magnetite, we collected samples spaced 5–14 m apart from 225 to 177.5 m depth in core BK2, refining our sample spacing to 0.5 m in regions of interest. In this stratigraphic interval, there is only a single magnetitite layer, which is a thin (< 1 cm) layer at 197 m depth.

Using a hand-held XRF device, we determined the locations of high bulk rock P_2_O_5_ concentrations through the lowest part of the UZc stratigraphy in drill core BK1. We found three localised regions containing cumulus apatite: the lowest of these three zones, at 1645–1640 m depth in BK1, was reported by Cawthorn and Walsh (1988) although their sampling was not sufficiently detailed to detect the other two apatite-bearing layers. The lowest of these two previously unreported zones occurs between 1607 and 1594 m depth in BK1, whereas the other has its base between 1573 and 1573.5 m depth and its top between 1544.08 and 1543.96 m depth. For this study, we concentrated on this latter apatite-rich horizon, and collected a detailed series of samples immediately below both the arrival and the disappearance of abundant (presumed cumulus) apatite.

## Petrography

### Lower Main Zone

**Fig. 8:**
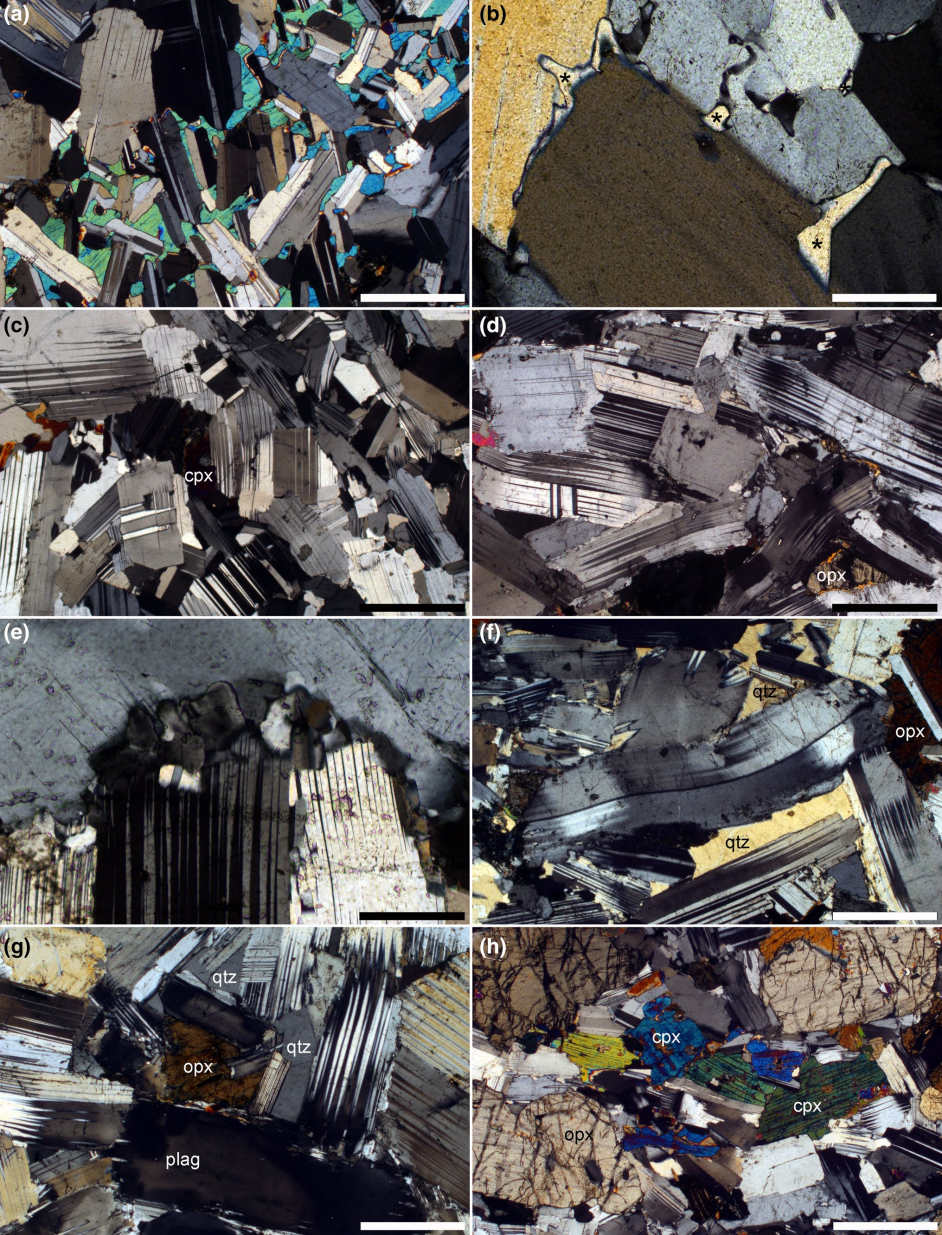
Photomicrographs of cumulates in the Lower Main Zone, all under crossed polars. All sample numbers refer to depth in core SL12 in metres. **a** 1557.9. An extended clinopyroxene oikocryst encloses relatively undeformed plagioclase with a weak preferred orientation forming a foliation. Scale bar is 1 mm long. **b** 1539.7. Irregular interstitial pockets filled with quartz. All quartz grains (marked with an asterisk) in this field of view are in optical continuity. Scale bar is 200 μm long. **c** 1533.8. Plagioclase primocrysts with a low apparent aspect ratio, forming a weak foliation. The grains contain deformation twins and commonly have distorted lattices. Note the loss of well-defined low-index growth faces and the irregular grain boundaries indicative of some dynamic recrystallization. The field of view contains an interstitial clinopyroxene grain (cpx) in extinction. Scale bar is 1 mm long. **d** 1403.4. Deformed plagioclase primocrysts with an average apparent aspect ratio higher than in **c**, and a relatively high density of deformation twins with abundant evidence of distorted lattices. Scale bar is 1 mm long. **e** 1425.55. Grain boundary between two deformed plagioclase grains is decorated with neoblasts formed during dynamic recrystallization. Scale bar is 200 μm long. **f** 1248.31. Abundant interstitial quartz (qtz) forms extensive areas in optical continuity. Note the complete absence of evidence of deformation in the quartz in comparison to the significant lattice distortion in the bounding plagioclase primocrysts. Scale bar is 1 mm long. **g** 1425.55. Cumulate comprising primocrysts of plagioclase and orthopyroxene (opx) with interstitial undeformed quartz. The crystal labelled “plag” contains well-developed compositional zoning. Scale bar is 1 mm long. **h** 1377.15. Cumulate containing primocrysts of plagioclase and large equant primocrysts of orthopyroxene, together with primocrysts of clinopyroxene. The cumulus status of the clinopyroxene is evident by its compact morphology, preferred alignment of elongate grains parallel to the weak foliation and the presence of multiple individual grains. Scale bar is 1 mm long

In the lowest region of our sample traverse through Lower Main Zone, the cumulates comprise anorthosites with highly skeletal oikocrysts of both ortho- and clinopyroxene (Fig. 8a). The oikocrysts of orthopyroxene become gradually more compact up-section until they form clearly distinct primocrysts at 1514 m depth. Small amounts of interstitial quartz are present, filling elongate pockets between plagioclase grains (Fig. 8b). At the base of our sample traverse, the plagioclase grains are equant with a low average apparent aspect ratio (Fig. 8c). A weak crystallographic preferred orientation is present, forming a foliation. Grains show evidence for some dislocation creep, with tapered deformation twins and lattice distortion (Fig. 8c). Grain boundaries between plagioclase grains are irregular, with interpenetration of grains, establishment of 120° junctions and little preservation of low-index growth faces (Fig. 8c).

Between 1540 and 1530 m depth in the core, the average apparent aspect ratio of the plagioclase increases, although the strength of the fabric (as estimated from thin section) does not change. The amount of lattice distortion in plagioclase and pyroxene increases significantly over this stratigraphic interval, with an increase in the number of tapered twins in plagioclase (Fig. 8d), an increase in the irregularity of the boundaries separating plagioclase grains, coupled with the appearance of neoblasts decorating the grain boundaries (Fig. 8e). In contrast to the lower parts of our traverse, in which plagioclase is the only mineral exhibiting evidence for dislocation creep, both ortho- and clinopyroxene grains have distorted lattices and undulose extinction above this level in the stratigraphy. The amount of deformation remains constant through much of the remaining sampled stratigraphic interval, but gradually decreases above ~ 1300 m depth to levels similar to those seen at the bottom of the sample traverse by ~ 1000 m depth.

In association with the onset of microstructural modification by dislocation creep at 1540–1530 m depth in the core, the amount of interstitial quartz in the cumulates increases to several vol.% (Fig. 8f), and the plagioclase is commonly compositionally zoned (Fig. 8g). Quartz forms extensive single crystals occupying pockets bounded by euhedral growth faces of plagioclase primocrysts, and is almost entirely undeformed (with only rare evidence of undulose extinction) even when adjacent to strongly deformed plagioclase grains (Fig. 8f, g).

Clinopyroxene is always less abundant than orthopyroxene. It is interstitial at the base of our sample traverse with the exception of two samples at 1440.7 and 1432.92 m. (As these are bracketed by samples at 1454 and 1425 m which contain interstitial clinopyroxene, the stratigraphic thickness of this interval containing clinopyroxene primocrysts must be between 7 and 25 m). The primocrystic status of the clinopyroxene is evident not from an increase in modal abundance but from a compact and elongate shape [long axis parallel to (001)], a preferred orientation with the long axis parallel to the weak fabric created by the plagioclase, and the presence of grain clusters (Fig. 8 h). Some of the grains have interstitial overgrowths. Clinopyroxene primocrysts are absent above 1432.92 m and do not re-appear until 1377.15 m depth in the core: all our samples overlying this horizon contain clinopyroxene primocrysts. The two samples immediately underlying the sample at 1377.15 m (1377.85 and 1378.3 m) contain clinopyroxene with a habit intermediate between oikocrysts and primocrysts. The first sustained appearance of cumulus clinopyroxene in this core thus occurs ~ 190 m above the base of the Merenksy Reef and ~ 169 m above the base of the Giant Mottled Anorthosite.

### Upper Zone

**Fig. 9:**
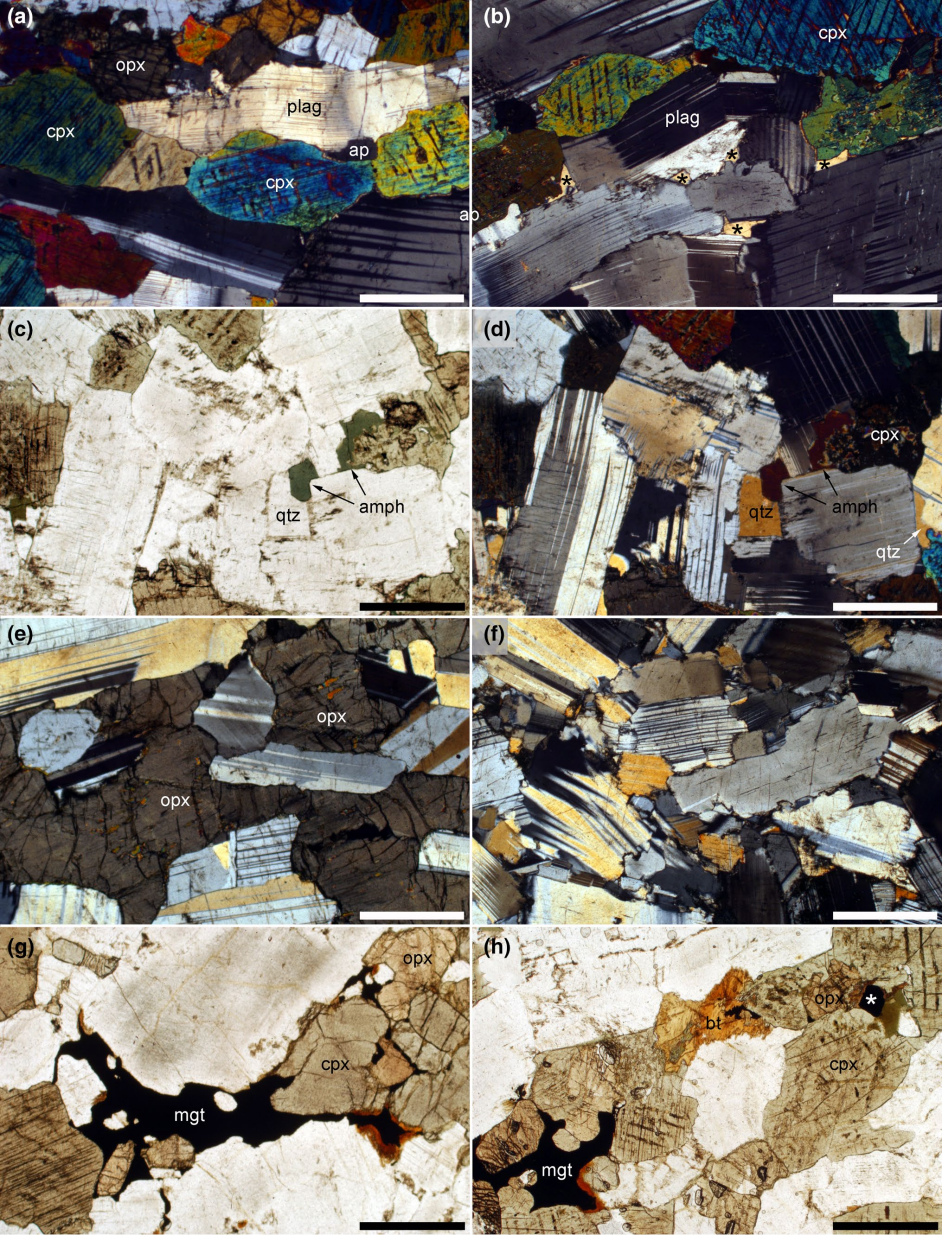
Photomicrographs of cumulates from the drill core BK2. All sample numbers refer to the depth in the core in metres. **a** Sample 227. Cumulate containing primocrysts of clinopyroxene (cpx), othropyroxene (opx) and plagioclase. Note the elongation of the clinopyroxene grains parallel to the weak foliation defined by the (commonly deformed) plagioclase. Scale bar is 1 mm long. **b** Sample 196. Interstitial and undeformed quartz (marked by asterisks) forms extensive groups of grains in optical continuity. Note the irregular grain boundaries of the plagioclase, with the loss of low-index growth faces. Scale bar is 1 mm long. **c** Sample 219. Green amphibole (amph) forms rims and overgrowths on clinopyroxene primocrysts, particularly where adjacent to interstitial quartz. Plane polarised light. Scale bar is 1 mm long. **d** Sample 219. The same field of view as **c** under crossed polars. Note the deformation in the plagioclase, manifest by deformation twins, distorted lattices and irregular grain boundaries. Scale bar is 1 mm long. **e** Sample 219. Oikocrst of orthopyroxene enclosing relatively undeformed plagioclase primocrysts. Scale bar is 1 mm long. **f** Sample 196. Evidence of deformation by dislocation creep of the plagioclase, with deformation twins, distorted crystal lattices and irregular grain boundaries. Scale bar is 1 mm long. **g** Sample 211. Interstitial magnetite (mgt) filling spaces between primocrysts of plagioclase, orthopyroxene and clinopyroxene. Scale bar is 1 mm long. **h** Sample 196. This field of view shows apparently interstitial magnetite (labelled “mgt”) as well as a single isolated equant grain (labelled with an asterisk) of cumulus magnetite. Interstitial biotite (bt) is also present. Scale bar is 1 mm long

In the lower part of UZ, as sampled by drill core BK2, the cumulus assemblage is plagioclase, orthopyroxene and clinopyroxene (Fig. 9a), with interstitial magnetite, biotite, apatite and quartz (Fig. 9b). The clinopyroxene locally has rims of green amphibole, most commonly in close association with interstitial quartz (Fig. 9c, d). Both clino- and orthopyroxene generally form equant compact grains but locally the orthopyroxene grains enclose plagioclase (Fig. 9e). The plagioclase is relatively equant, with localised, but minor, evidence of dislocation creep (distorted crystal lattice, discontinuous deformation twins, and irregular grain boundaries) (Fig. 9f). The extent of deformation recorded by the plagioclase varies non-systematically with stratigraphy, with some samples apparently almost unaffected: there is no obvious pattern between the extent of deformation and other microstructural features such as grain size, strength of fabric and modal mineralogy. In contrast to the drill core SL12 from Lower Main Zone, in which the pyroxenes are also deformed, in the lower part of UZ sampled here no minerals other than plagioclase display evidence of crystal plastic deformation, and nowhere do we see deformation as strong as that described in Lower Main Zone. The generally small and scattered grains of (undeformed) interstitial quartz (modes < 1 vol.%) locally form oikocrysts of a similar overall dimension to the cumulus plagioclase grains which bound the quartz-filled pockets.

Magnetite forms irregular interstitial patches, at low modal proportions, throughout the stratigraphic section sampled (Fig. 9g), with the exception of a thin magnetitite layer at 197 m. In the upper part of our section (from 196 m upwards), it also forms isolated rounded or euhedral grains located at interphase grain boundaries or enclosed by pyroxene or plagioclase (Fig. 9h). We interpret this habit as cumulus, despite the uniformly low modal abundance of magnetite. The first appearance of these isolated euhedral grains is not well defined, with very rare (0–2 grains per section) small examples present at 200 m depth.

**Fig. 10:**
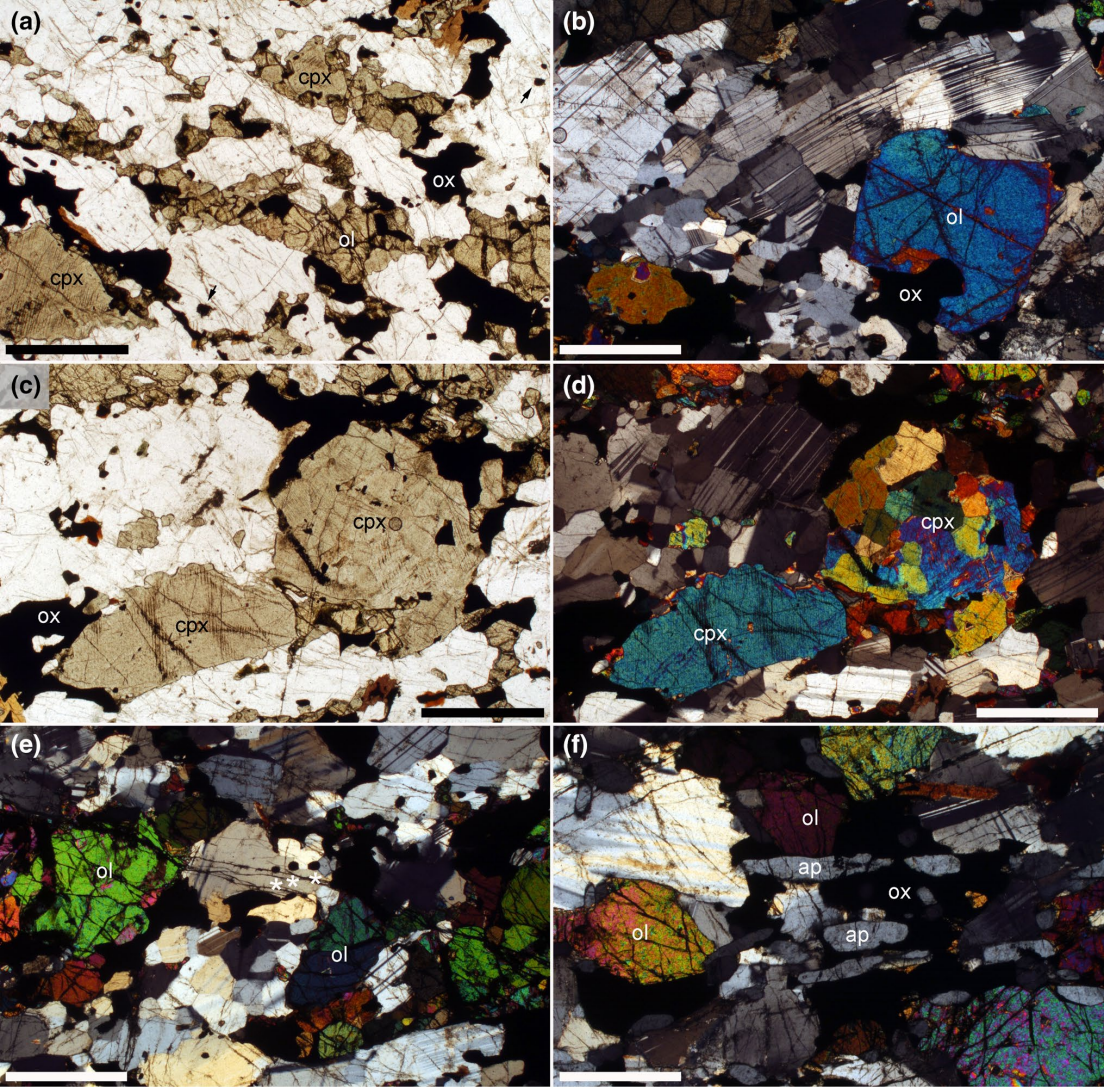
Photomicrographs of the upper part of Upper Zone. All sample numbers refer to the depth in metres in drill core BK1. **a** Sample 1574. A foliation is manifested by the alignment of elongate primocrysts of plagioclase and clinopyroxene, together with elongate clusters of olivine (ol), clinopyroxene (cpx) and magnetite (ox). Note the biotite associated with the magnetite at the top right of the image. Scale bar is 1 mm long. **b** Sample 1574. Large crystals of plagioclase are commonly deformed, while finer-grained regions of plagioclase have a granular microstructure (see region to the left of the labelled olivine primocryst). Scale bar is 1 mm long. **c** Sample 1574. Clinopyroxene grain size is bimodal, with the smaller grains commonly forming large clusters. Plane polarised light. Scale bar is 1 mm long. **d** Sample 1574. The same area as **c** under crossed polars, in which the polycrystalline nature of the clinopyroxene cluster is evident. Note the granular microstructure in the cluster, indicative of sub-solidus textural equilibrium. Scale bar is 1 mm long. **e** Sample 1574.5. Low aspect ratio plagioclase, with a granular microstructure locally. White asterisks lie below examples of isolated cumulus grain of magnetite. Scale bar is 1 mm long. **f** Sample 1549.3. Cumulus apatite (ap) forms elongate grains parallel to the foliation, commonly associated with magnetite. Scale bar is 1 mm long

Below the arrival of cumulus apatite in the upper part of Upper Zone, as sampled by drill core BK1, clinopyroxene, olivine, plagioclase and magnetite are primocryst phases, with small amounts of interstitial biotite and apatite (Fig. 10a, b). The interstitial biotite is invariably associated with magnetite (Fig. 10a). A foliation is formed by the alignment of large elongate clinopyroxene and plagioclase primocrysts, together with the alignment of elongate clusters of olivine and clinopyroxene (Fig. 10a). Clinopyroxene has a bimodal grain size although some of the fine-grained polycrystalline pyroxene clusters are of a similar size to the large single crystals (Fig. 10c, d). Plagioclase grain size is also bimodal, with the large grains commonly showing some evidence of deformation by dislocation creep (lattice distortion and tapered twins), while regions of finer-grained plagioclase commonly have a granular microstructure (Fig. 10b, e). There is no sign of the irregular grain boundaries and neoblasts described from the lower parts of the stratigraphy: in contrast, the microstructures indicate significant approach to sub-solidus textural equilibrium in fine-grained monomineralic regions, with localised development of granular microstructure (Fig. 10b, d, e).

The arrival of cumulus apatite is marked by the appearance of abundant elongate grains which are generally aligned parallel to the foliation and are commonly spatially associated with magnetite. Although magnetite is often apparently interstitial, its cumulus status is indicated by isolated equant grains on grain boundaries or enclosed by single crystals of other primocryst phases (Fig. 10e).

## Results

### Clinopyroxene-in

**Fig. 11:**
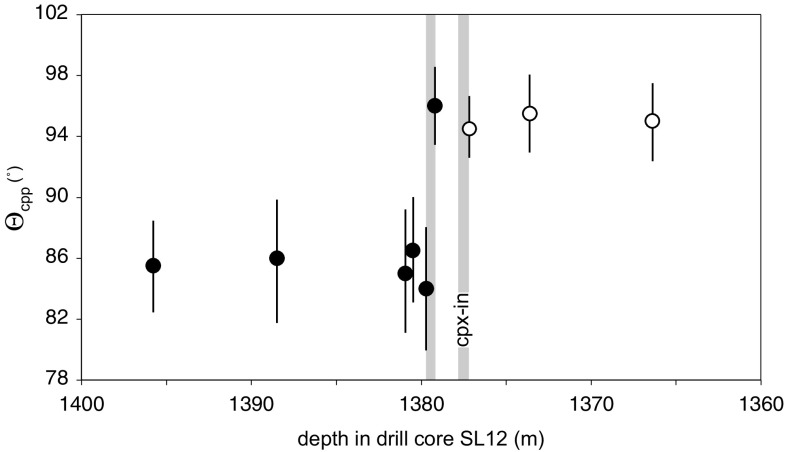
The stratigraphic variation of Θ_cpp_ in the vicinity of clinopyroxene-in, as measured in the Lonmin core SL12. The uncertainties are calculated according to the method of Stickels and Hücke (1964). Black dots show Θ_cpp_ in samples without cumulus clinopyroxene, while open symbols refer to samples with cumulus clinopyroxene. The grey vertical bars give the stratigraphic uncertainties (as a consequence of sample spacing) for the position of the step-change in Θ_cpp_ and the arrival of cumulus clinopyroxene

The stratigraphic variation of Θ_cpp_ in the vicinity of the first sustained appearance of cumulus clinopyroxene in core SL12 is shown in Fig. 11, with the results presented in Table 1. The step-change in Θ_cpp_, at which it increases from values in the range 82.5°–86.5° to values in the range 94.5°–98°, is bracketed to within 0.5 m of stratigraphy, occurring between the two samples at 1379.2 and 1379.5 m depth. The appearance of unambiguously cumulus clinopyroxene occurs at 1377.15 m depth in the core, with unambiguously intercumulus clinopyroxene below 1378.3 m: the offset between the two markers denoting the top and bottom of the mush at the moment the bulk magma saturated in clinopyroxene is therefore no more than ~ 2 m.

Notably, Θ_cpp_ in the sample at 1432.95 m depth in core SL12 is 94.5°, consistent with the cumulus status of clinopyroxene in this sample. We did not sample sufficiently closely in this region of temporary saturation of the bulk magma in clinopyroxene to use this as a further constraint on mush thickness.

### Magnetite-in

**Fig. 12:**
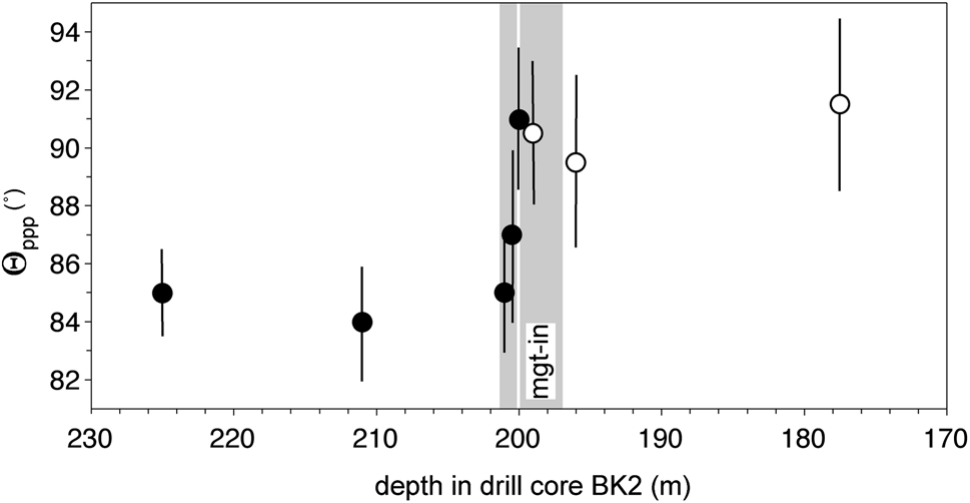
The stratigraphic variation of Θ_ppp_ in the vicinity of magnetite-in, as measured in the Bierkraal core BK2. The uncertainties are calculated according to the method of Stickels and Hücke (1964). Black dots show Θ_ppp_ in samples without cumulus magnetite, while open symbols refer to samples with cumulus magnetite (although note that the cumulus status in the sample at 199 m is not certain). The grey vertical bars give the stratigraphic uncertainties (as a consequence of sample spacing) for the position of the step-change in Θ_ppp_ and the arrival of cumulus magnetite

The stratigraphic variation of Θ_ppp_ in the vicinity of the lowest magnetitite layer in core BK2 is shown in Fig. 12, with the results presented in Table 1. The stratigraphic position of the arrival of cumulus magnetite is not well constrained: although it is certainly a cumulus phase at 197 m (the position of the magnetitite layer), cumulus magnetite may be present at 200 m. The step-wise increase in Θ_ppp_ associated with the arrival of cumulus magnetite occurs between the samples at 200 and 201 m depth. These constraints place lower and upper bounds on mush thickness of 1 and 4 m, respectively: the upper bound is reduced to 3.7 m if we take the 24° dip of the layering into account.

Interestingly, the median dihedral angle below magnetite-in is lower (~ 84°) than that in the cumulates above clinopyroxene-in (~ 94°), despite the total number of liquidus phases being higher: the change in dihedral angle through the stratigraphy is therefore not a simple staircase as is shown for the upper parts of the Rustenburg Layered Suite in Fig. 3. The actual value of dihedral angle is a complex function of cooling history via a dominant control by the relative rates of growth of the various plagioclase growth faces: the growth rates of the second mineral phase (either pyroxene or olivine) are likely to exert a secondary control. Our data suggest that the median dihedral angle decreases from ~ 94° to ~ 84° through much of Main Zone, although the cause of this is not clear.

### Apatite-in

**Fig. 13:**
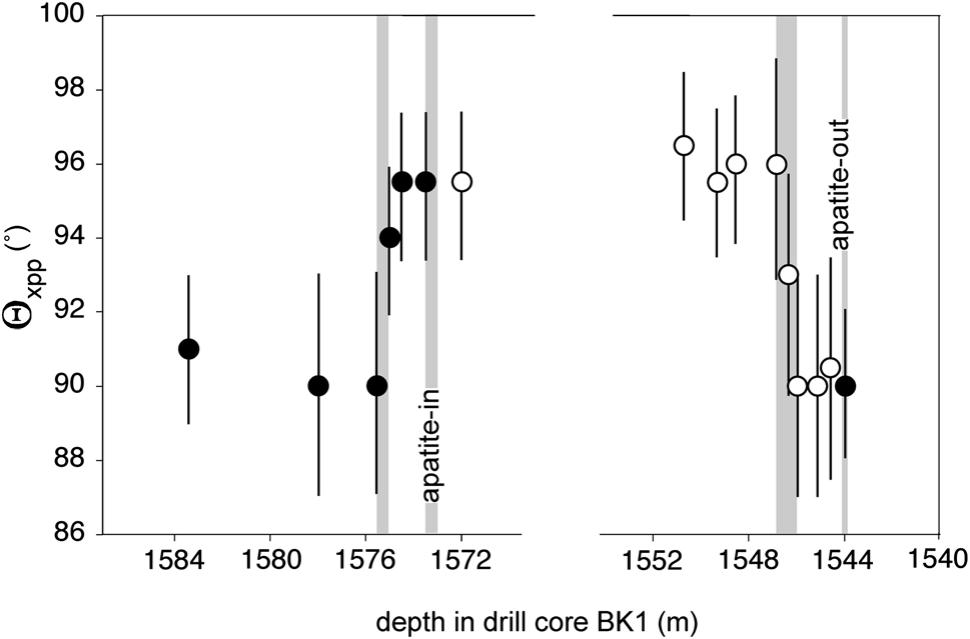
The stratigraphic variation of Θ_xpp_ in the vicinity of the third-lowest localised horizon containing cumulus apatite, as measured in Bierkraal core BK1. Note the discontinuity in the x-axis. The uncertainties are calculated according to the method of Stickels and Hücke (1964). Black dots show Θ_xpp_ in samples without cumulus apatite, while open symbols refer to samples with cumulus apatite. The grey vertical bars give the stratigraphic uncertainties (as a consequence of sample spacing) for the position of the step-changes in Θ_xpp_ and the arrival and disappearance of cumulus apatite

The stratigraphic variation of Θ_xpp_ in the vicinity of a localised appearance of cumulus apatite in core BK1 is shown in Fig. 13, with the results presented in Table 1. The position of the base of this apatite-bearing horizon is known within 50 cm, whereas that of its top is known within 12 cm. The step-wise increase from ~ 90° to ~ 96° in Θ_xpp_ associated with the arrival of cumulus apatite occurs between the two samples at 1575.54 and 1575.0 m depth, limiting the mush thickness in this region to a maximum of 2.5 m (which, if we take the dip of the layering into account, reduces to 2.3 m). The step-wise decrease, from ~ 96° to ~ 90° in Θ_xpp_ associated with the disappearance of cumulus apatite occurs between the two samples at 1545.97 and 1546.8 m (with one sample with an intermediate value of Θ_xpp_ at 1546.3 m), limiting the mush thickness to a maximum of 2.8 m (or 2.6 m if we account for the dip of the layering).

## Discussion

### The thickness of the floor mush in the Rustenburg Layered Suite

The thickness of the mush on the floor of the magma chamber in which the Rustenburg Layered Suite crystallised, as constrained by the offset between step-changes in median dihedral angle and the associated change in liquidus assemblage, was of the order of a few metres, with a maximum of ~ 4 m possible at magnetite-in. However, we have only determined mush thickness at three horizons in the Bushveld stratigraphy: it is possible that the mush was thicker at different times (or in different places) than those examined here. Recent work on the Skaergaard Intrusion suggests that the mushy layer may be transiently thick, due to collapse of mushy layers on a nearby vertical wall (Holness et al. 2017a). In the context of the Bushveld, transiently thick mushy layers might be generated by settling of phenocrysts from incoming magma (e.g. Mondal and Mathez 2007), or by the slumping of large areas of poorly consolidated crystal mush on a sloping floor (e.g. Maier et al. 2013). Detecting stratigraphic intervals characterised by a thick mush requires many more data points than the few presented here: while this is possible in that part of UZ characterised by the cyclic appearance and disappearance of cumulus apatite (Tegner et al. 2006), constraints on mush thickness in regions of stratigraphy far from horizons marking the arrival or disappearance of liquidus phases can only be obtained using detailed field observations.

Our determination of < 4 m mush thickness is similar to the thickness determined across much of the floor of the Skaergaard magma chamber at the moment of saturation of the bulk magma in apatite (Holness et al. 2017a). Such a shallow mush depth is consistent with the commonly observed evidence for a rigid mush underlying the very dense oxide layers, the presence in the cumulate stratigraphy of abundant potholes that truncate igneous layering (e.g. Latypov et al. 2015), and the absence of thick (≫ 10 m) packets of distorted and slumped layering (e.g. Fig. 7).

### Implications of results for magma chamber processes

Our measurements of mush thickness contrast with the recent suggestions of Hayes et al. (2017) who argued, on the basis of geochemistry and microstructures, for the repeated downwards infiltration of replenishing magma through tens of metres of mush in Main Zone. Such infiltration would require high porosity over this distance, since the microstructural evidence they present is not consistent with interfacial energy-driven infiltration of sub-solidus cumulates (e.g. Holness et al. 2012b). The evidence of Hayes et al. (2017) relates to an upward increase in the Mg# in mafic minerals associated with plagioclase of a constant An content: an alternative explanation for this feature was offered by Cawthorn (2015) who suggested it is due to the trapped liquid shift effect.

Our conclusion that the mush was thin places fundamental constraints on the processes that can occur in the mush. For example, compositional convection in a basaltic mush can only occur if the mush thickness exceeds several hundred metres (Tait and Jaupart 1992). Similarly, although many studies of mafic layered intrusions suggest that the crystal mush compacts under its own weight, expelling the interstitial liquid and promoting the formation of adcumulates (e.g. Irvine 1980; Sparks et al. 1985; Shirley 1986; Tharp et al. 1998; Meurer and Meurer 2006; Tegner et al. 2009; McKenzie 2011), the effective operation of this process in a basaltic system requires a mush thickness of order 300 m (McKenzie 2011). Our conclusions suggest that the mush forming the Rustenburg Layered Suite was insufficiently thick to permit compositional convection. Similarly, several metres of mush is unlikely to be sufficient to drive compaction for typical gabbroic compositions, although the published calculations do not consider the presence of metre-scale layers of oxide minerals interlayered with much lower density silicate-rich layers.

### What caused the deformation in the Rustenburg Layered Suite?

We have recorded abundant evidence of bent and distorted crystal lattices, irregular grain boundaries, and neoblasts formed during syn-deformational recovery: these features are all indicative of deformation by dislocation creep. The most commonly cited cause of deformation in cumulates is gravitationally-driven viscous compaction, which is suggested to involve either dislocation creep (e.g. McKenzie 2011) or diffusive processes such as dissolution–reprecipitation that result in the complete recrystallization of primary igneous microstructures (e.g. Boorman et al. 2004). Although we have presented evidence in support of a thin mushy layer that is unlikely to have exerted a significant load, we need to consider other indicators to establish whether or not the observed deformation may have been caused by viscous compaction.

The samples with the best developed evidence for dislocation creep are those in the drill core through Lower Main Zone, in which the cumulus assemblage is dominated by plagioclase and pyroxene. This deformation occurred while the mush contained residual liquid, as the interstitial quartz is not deformed and therefore must have crystallised after deformation had ceased. It is unlikely that sufficient pressure to account for the observed deformation could have been exerted by an overlying gabbroic mush of only a few metres thickness. Importantly, the extent of deformation varies within our sampled 500 m stratigraphic interval through Lower Main Zone, with almost no evidence of dislocation creep in the lower few tens of metres, and a gradually declining extent of deformation in the upper three hundred metres of the interval. The mineral mode does not change significantly over this 500 m of stratigraphy, and the consistent measured mush thickness of a few metres in three widely spaced places in the Bushveld stratigraphy suggests mush thickness may also not have varied significantly through the 500 m interval sampled by the Lonmin core: it is therefore unlikely that gravitational loading by the mush would have varied sufficiently to account for the difference in strength of deformation.

The mode of interstitial quartz is lowest in the lower few tens of metres of the sampled interval and highest in the samples containing the most deformed plagioclase, contrary to what would be expected if the volume of interstitial liquid had been reduced during viscous deformation. Furthermore, if we were to unbend the most distorted plagioclase grains, it is unlikely to have much effect on the volume of the intervening porosity (e.g. Figs. 8f, 9b). These observations, although qualitative, suggest that the observed viscous deformation did not have much effect on the extent to which late-stage liquids were expelled from Lower Main Zone cumulates.

Cawthorn and Walsh (1988) argue that the amount of interstitial liquid in UZ in the vicinity of apatite-in was in the region 1–6%, and thus that the cumulates in this region of the intrusion are adcumulate. These rocks show little evidence for dislocation creep, with only the largest plagioclase grains recording lattice distortion. Although the fine grain size and granular microstructure might suggest overprinting of a primary igneous fabric by dynamic recrystallization during dislocation creep, there is no sign of neoblasts on grain boundaries or lattice distortion in minerals other than plagioclase. We conclude that these adcumulates experienced almost no viscous deformation by dislocation creep.

Boorman et al. (2004) argued that viscous compaction, achieved by diffusive processes such as dissolution–reprecipitation and recrystallization, was a dominant process in Critical and Lower Zone. We do not have detailed geochemical information for our samples that can be used to demonstrate the preservation of original growth faces and compositional zoning (as described by Holness et al. 2017b), and therefore cannot make a full assessment of the extent to which diffusive processes may have acted in the Rustenburg Layered Suite. However, the dominant shape of plagioclase in the samples we examined is one defined by flattening parallel to (010), typical of growth of plagioclase from magma. The only exception to this is the granular microstructure observed in monomineralic regions, particularly in the vicinity of apatite-in. Similarly, cumulus orthopyroxene grains have shapes that are unchanged from those likely to have been created by growth from magma. The clearly preserved evidence for super-solidus deformation by dislocation creep (e.g. Fig. 8) demonstrates that complete recrystallization did not occur once the dislocation creep had ceased, and we argue that it is highly unlikely that the dislocation creep could have post-dated an event resulting in the complete recrystallization of a primary igneous microstructure due to compaction by diffusive processes.

That some compaction, by either dislocation creep or diffusive processes, may have occurred in the samples we examined is certainly possible. The localised distortion of the UG1 layering at Dwars River (Fig. 6a) is consistent with some compaction of the cumulate pile. However, this kind of feature is unusual, despite the base of thick oxide layers being the one place where we would expect the greatest extent of gravitationally-driven viscous compaction and disruption of underlying poorly consolidated (and therefore weak and deformable) material by the formation of load casts. We suggest, therefore, that viscous compaction was not a generally significant process in the Rustenburg Layered Suite, consistent with our conclusion that the mushy layer was generally only a few metres thick.

The question then remains as to the cause of the deformation recorded in the samples we examined. We have described how the extent of disruption of the layering varies significantly over short stratigraphic distances (as observed at Dwars River, for example): such behaviour is suggestive of brief episodic events which destabilise the mush [such as might be expected during updoming affecting the Dwars River region (Clarke et al. 2005)]. We suggest that the well-defined onset of significant dislocation creep near the base of our traverse through Lower Main Zone marks the start of a more regional deformation event.

The overall shape of the Bushveld is consistent with significant isostatic sinking (c.f. Cawthorn et al. 1998; Cawthorn and Webb 2001) and it has been argued that this was syn-magmatic, leading to significant collapse and slumping of poorly consolidated mush (Maier et al. 2013). Although paleomagnetic studies indicate that the Rustenburg Layered Suite cooled in an essentially horizontal position (Letts et al. 2009), it is possible that local earthquake-triggered slumping might have occurred on sufficiently shallow slopes to be consistent with the paleomagnetic data (Maier et al. 2013). Additionally, the base of the Main Zone is located close to the level where a major addition of magma is thought to have occurred, and this may have triggered significant readjustments in the floor while the lowest few hundred metres of Main Zone accumulated, leading to the observed deformation.

The absence of deformed interstitial quartz demonstrates that the mush must have become rigid and undeformable by the time the remaining liquid crystallised quartz. The quartz mode is generally low (a few vol.%) in these rocks (and in the lower part of UZ, where similar undeformed interstitial quartz is present), and it is possible that super-solidus deformation continued after the complete dihedral angle population had been created. This means that the actual thickness of deforming super-solidus mush would have exceeded the ~ 2 m distance between the top of the mush and the horizon at which all dihedral angles were formed. We, therefore, envisage an extended deformation event involving the continuous slumping and deformation of the upper parts of a continuously growing mush, with deformation occurring down to depths greater than a few metres but not extending into the fully solidified cumulates. The thermal model of Cawthorn and Walraven (1998) suggests that the accumulation rate of Lower Main Zone was ~ 5 cm year^−1^, suggesting that the deformation event lasted of the order a few thousand years. The gradual decline in the strength of deformation suggests that the deformation event similarly became less important with time.

The presence of deformed plagioclase in UZ suggests that similar regional-scale deformation may have occurred later in the history of the Rustenburg Layered Suite, although a full assessment can only be made by comparing the extent of deformation in magnetite-poor regions compared to anorthosites immediately underlying metre-scale magnetitite layers.

### Adcumulate formation in the Rustenburg Layered Suite

Our conclusions that viscous compaction was insignificant in the Rustenburg Layered Suite, and that the thin mush precluded compositional convection, means that adcumulate formation must have involved different processes. The thin mush is consistent with a predominance of in situ crystallisation and efficient mass exchange with the overlying bulk magma. We suggest that adcumulates formed during primary crystallisation on the magma chamber floor, resulting in enhanced growth of primocrysts, as initially suggested by Wager et al. (1960), and specifically by Cawthorn and Walsh (1988) for the Rustenburg Layered Suite. Such a process may be enhanced by mass transport in a temperature gradient (e.g. Walker et al. 1988), although it is not known how effective this process might be in a large, slowly cooled, magma body in which any temperature gradient is likely to be very small.

## Conclusions

The thickness of the mush in the Rustenburg Layered Suite of the Bushveld Complex, defined by the distance from the top of the mush to the horizon at which all three-grain junctions involving two grains of plagioclase and a mafic phase (pyroxene or olivine) are fully formed, is unlikely to have exceeded a few metres. This result has fundamental implications for our understanding of mush behaviour. In particular, processes that require a thick mush, such as viscous compaction (of relatively oxide-poor lithologies), compositional convection, and those relying on large-scale migration of reactive fluids through extensive mush layers, such as those advocated for the formation of PGE deposits (Boudreau and Meurer 1999) or cryptic layering of cumulates (Hayes et al. 2017), are unlikely to be significant in the development of the Rustenburg Layered Suite unless there are numerous episodes in which the mush is transiently thick (due to slumping or replenishment by crystal-rich magma). Hence, the widely preserved evidence for super-solidus ductile deformation by dislocation creep is likely to be a result of large-scale lateral deformation events, rather than simple gravitational loading of the crystal pile. The timing of the deformation recorded by the cumulates is constrained only by the presence of small volumes of undeformed late-stage magmatic quartz: since all dihedral angles (Θ_cpp_, Θ_ppp_ and Θ_xpp_) were likely to have been formed before the last drops of liquid crystallised as quartz it is possible that deformation involved a thicker packet of cumulates than a few metres.

These preliminary conclusions should be tested by a more detailed investigation of the extent and style of deformation in the Rustenburg Layered Suite, linking microstructures to the location of thick oxide layers and mapping out regional-scale variations in deformation to examine the role of km-scale events.

## Electronic supplementary material

Below is the link to the electronic supplementary material.

Supplementary material 1 (XLS 88 kb)
